# Stress and cardiometabolic manifestations among Saudi students entering universities: a cross-sectional observational study

**DOI:** 10.1186/1471-2458-14-391

**Published:** 2014-04-23

**Authors:** Nasser M Al-Daghri, Abdulaziz Al-Othman, Omar S Al-Attas, Khalid M Alkharfy, Majed S Alokail, Abdulmajeed Albanyan, Shaun Sabico, George P Chrousos

**Affiliations:** 1 Prince Mutaib Chair for Biomarkers of Osteoporosis, Biochemistry Department, College of Science, King Saud University, P.O. Box, 2455, Riyadh 11451, Kingdom of Saudi Arabia; 2 Biomarkers Research Program, Biochemistry Department, College of Science, King Saud University, Riyadh 11451, Kingdom of Saudi Arabia; 3 Center of Excellence in Biotechnology Research Center, King Saud University, Riyadh 11451, Saudi Arabia; 4 College of Applied Medical Sciences, King Saud University, Riyadh 11451, Kingdom of Saudi Arabia; 5 Clinical Pharmacy Department, College of Pharmacy, King Saud University, Riyadh 11451, Kingdom of Saudi Arabia; 6 Preparatory Year, King Saud University, Riyadh 11451, Kingdom of Saudi Arabia; 7 First Department of Pediatrics, Athens University Medical School, Athens 11527, Greece

**Keywords:** Stress, Cardiometabolic clustering, Saudi, Students, College life, Perceived stress

## Abstract

**Background:**

In this observational study, we aimed to see whether transition in Saudi students entering university life could be a breeding stage for cardiometabolic risk factor emergence and clustering.

**Methods:**

A total of 1878 apparently healthy Saudi students of the Preparatory Year, King Saud University, Riyadh, KSA (1112 men and 766 women) spanning 2 academic years were included. They were divided into 2 groups based on the validated perceived stress test (PST). Anthropometrics were obtained and fasting blood samples were collected for measurement of fasting blood glucose and a lipid profile.

**Results:**

PST score (>27) considered indicative of stress was noted in 44.4% of students. The prevalence of this score was higher in women than in men (49.7% versus 40.7%). The prevalence of obesity, hypertension and dyslipidemia was significantly higher in men than women (p < 0.01), and this was even more apparent among stressed men, who had a significantly higher prevalence of all the above cardiometabolic factors than the non-stressed ones (p < 0.01).

**Conclusion:**

Perceived stress is alarmingly high among Saudi students entering universities. This study sheds light on the social responsibility of universities in promoting a healthy lifestyle, particularly in this age group, when exposure to different kinds of stressors may result in body weight and metabolic changes.

## Background

Over the last decades, the Kingdom of Saudi Arabia has experienced a great deal of industrialization. This, together with Western influences, brought urbanization and lifestyle change. The impact of these changes is now evident, and its disadvantages abound, specifically its implications in increasing the burden of chronic non-communicable diseases [1]. Obesity is the by-product of the interplay between genetic and environmental factors. In general, over-nutrition and sedentary lifestyle are considered two major modifiable risk factors for the progression of both adult and childhood obesity. Metabolic syndrome (MetS), which is the clustering of cardiovascular risk factors, including obesity, hypertension, insulin resistance and dyslipidemia, is highly prevalent in both Saudi adults and children [2,3]. While obesity and MetS have been well studied in Saudi Arabia, prevention strategies have been limited. Adolescents and young adults have been largely ignored in most studies, despite the fact they too are exposed to stress, secondary to physical, hormonal, and social transitions.

Stress plays a significant role in the pathogenesis of obesity and MetS [4]. Psychosocial stress among adolescents in particular, is a risk factor for both unhealthy eating and obesity [5]. Students entering the university are vulnerable to weight gain, a phenomenon known as “freshman 15” (or freshman 5 in some) [5]. Studies on weight gain involving young adults in their first year of college have identified certain risk factors, which include, but are not limited to, disordered eating and dietary restraint for women [6], as well as body-building and troublesome relationships with parents for men [7]. Hence, this particular stage in a young adult’s life might be a critical developmental window for increased adiposity that requires the involvement of universities in framing prevention programs designed to curtail the weight gain attained by many vulnerable students.

In this study we chose young Saudi adults who are in transition to college life, in an attempt to determine whether sudden environmental changes translate to altered habits that predispose to obesity and other cardiovascular risk factors. To achieve this we will use a well-known tool in assessing global measure of perceived stress that has been used and validated from several studies [8-13]. This study could shed light on the social responsibility of universities and other learning institutions in promoting a healthy lifestyle, particularly in this vulnerable age group, when many transitions, both physical and psychosocial, are happening simultaneously. This study could also help reveal prospectively how this particular population handles stress and whether stress management could diminish cardiovascular risk factor progression.

## Methods

### Subjects

In this cross-sectional study, a total of 1878 apparently healthy Saudi students (1112 men and 766 women for the academic year 2010–2011) were invited to participate. This is a single-center study conducted at the Preparatory Year (PY) College of King Saud University (KSU), Riyadh, Saudi Arabia. KSU is a state university of KSA with different colleges including PY with almost 35000 students coming all over KSA and neighboring Gulf countries.

### Design questionnaire

Students answered a pre-designed questionnaire that was developed, pre-tested and validated (Table 1). The questionnaire has an internal consistency of 0.85 (Cronbach α co-efficient) and test-retest reliability during a short retest interval (several days) of 0.85 [14]. The questionnaire has several parts including: socio-demographic data, medical history, physical activity, sleep assessment, and a perceived stress test [13,14]. Perceived stress test scores are obtained by reversing the scores on four positive items, with a possible range of scores from 0 to 56 [13]. Ethics approval was obtained from the College of Medical Research Center (CMRC), KSU, Riyadh, Saudi Arabia. Written informed consents were required from participants prior to inclusion. Approval from the Dean of the Preparatory Year was also sought prior to commencement of studies.

**Table 1:** Percentages (%) of students’ responses in the perceived stress test

	**Never**	**Rarely**	**Sometimes**	**Usually**	**Always**
In the last month, how often have you…					
Been upset because of something that happened unexpectedly?	5.3	11.7	47.3	27.3	8.4
Felt that you were unable to control the important things in your life?	9.6	24.1	42.6	17.3	6.4
Felt nervous and “stressed”?	5.8	19.7	40.9	25.4	8.3
Dealt successfully with irritating life hassles?	2.7	9.0	43.5	37.3	7.6
Felt that you were effectively coping with important changes occurring in your life?	3.3	8.8	41.9	35.0	11.0
Felt confident about your ability to handle your personal problems?	3.8	7.9	29.9	38.0	20.4
Felt that things were going your way?	4.1	13.2	48.7	27.1	7.0
Found that you could not cope with all the things that you had to do?	13.8	29.8	41.1	12.2	3.2
Been able to control irritations in your life?	4.9	17.3	46.1	24.4	7.3
Felt that you were on top of things?	5.2	16.5	46.3	23.8	8.2
Been angered because of things that happened outside of your control?	4.7	16.0	42.3	26.4	10.7
Found yourself thinking about things that you have to accomplish?	3.2	6.0	25.0	39.2	26.6
Been able to control the way you spend your time?	6.4	21.5	45.2	21.1	5.8
Found difficulties were piling up so high that you could not overcome them?	6.9	24.5	48.2	15.8	4.6

Note: Data presented in percentage (%).

### Inclusion and exclusion criteria

Only consenting students from the Preparatory Year were included in the study. Students who refused to participate, those with underlying acute and chronic medical conditions that warranted immediate attention (e.g. asthma), and students who were handicapped were excluded from the study.

### Anthropometrics

Weight was recorded to the nearest 0.2 Kg using an international standard scale (Digital Person Scale, ADAM Equipment Inc., USA); height to the nearest 0.5 cm using the same scale. BMI was calculated as kg/m^2^, and classified as lean, overweight or obese, depending on BMI for age and gender for subjects below 18 years. Waist and hip circumferences were measured by non-stretchable tape measure, and recorded to the nearest 0.1 cm. Body fat composition was also assessed by Body Fat Analyzer (Biospace Inc., USA) to determine body fat percentage, lean mass and fluids distribution. All measurements were administered by well-trained research assistants of the Biomarkers Research Program (BRP) of KSU, Riyadh, KSA.

### Biochemical assessment

Blood was withdrawn in the morning after an overnight fast (>10 hours) and collected in non-heparinized test tubes by an assigned physician. Fasting serum glucose and lipid profile (Total, LDL- and HDL-cholesterol and triglycerides) were measured using routine laboratory procedures (Konelab, Finland). All biochemical estimations and storage of samples were carried out at BRP, KSU, Riyadh, KSA.

### Statistical analysis

Data were analyzed using SPSS 11.5 (Chicago, Illinois) and variables were expressed as mean ± standard deviation (SD) for continuous variables. Frequencies were presented in percentage (%). Student t-test was done to compare differences between 2 groups (with and without perceived stress). Bivariate correlations were done to determine associations between perceived stress and metabolic parameters of interest. P-value was considered significant at < 0.05.

## Results

A total of 1878 preparatory year students participated in this cross-sectional study spanning 2 academic years. Perceived stress was noted in 44.4% of students (N = 834, Table 2). Table 1 shows the percentage distribution of the student responses to the perceived stress test questionnaire, with the majority of responses falling under “sometimes”. Comparative analysis was performed among perceived stress versus non-perceived stress group (control) and revealed that those with perceived stress had a significantly higher total cholesterol (*p* = 0.03) and body muscle percentage (%) compared to controls. Furthermore, the perceived stress group had a significantly higher HDL-cholesterol (*p* = 0.028), basal metabolic rate (BMR) (*p* = 0.001), arm muscle circumference (AMC) (*p* = 0.02), body cell mass (BCM) (*p* = 0.001), intracellular (*p* = < 0.001), extracellular (*p* = 0.003), protein (*p* = 0.001) and mineral (*p* = < 0.001) measured values, body water (*p* = 0.002), soft lean mass (*p* = 0.001), and lean body mass (*p* = 0.001) than the controls (Table 2).

**Table 2:** General characteristics of the subjects studied (Cohort 2010–2011)

	**Control**	**Perceived stress**	**P value**
N	1044	834	
Gender (M/W)	659/385	453/381	<0.001
Age (years)	18.1 ± 2.8	18.2 ± 2.2	0.40
Body mass index (BMI) (kg/m^2^)	23.8 ± 5.4	23.8 ± 5.9	0.99
Waist circumference (cm)	80.3 ± 15.6	79.2 ± 17.2	0.18
Hip circumference (cm)	100.1 ± 14.4	99.8 ± 14.3	0.62
Sagittal abdominal diameter (SAD) (cm)	17.6 ± 6.9	17.3 ± 7.5	0.45
Total cholesterol (mmol/l)	4.2 ± 0.91	4.4 ± 0.97	0.03
Glucose (mmol/l)	5.1 ± 0.57	5.2 ± 0.58	0.64
HDL-cholesterol (mmol/l)	1.21 ± 0.32	1.24 ± 0.32	0.028
Triglycerides (mmol/l)	0.98 ± 0.48	0.99 ± 0.50	0.84
Basal metabolic rate (BMR)	1644.3 ± 279.3	1594.8 ± 280.5	0.001
Arm muscle circumference (AMC)	21.9 ± 3.1	21.5 ± 3.2	0.02
Body cell mass (BCM)	32.6 ± 7.5	31.2 ± 7.5	0.001
Intracellular measured value	23.4 ± 5.6	22.4 ± 5.3	<0.001
Extracellular measured value	11.1 ± 2.5	10.7 ± 2.4	0.003
Protein mass measured value	9.3 ± 2.1	8.9 ± 2.1	0.001
Mineral mass measured value	3.1 ± 0.56	2.9 ± 0.55	<0.001
Fat mass measured value	19.3 ± 11.4	19.5 ± 11.8	0.66
Body water	34.5 ± 7.7	33.2 ± 8.0	0.002
Soft lean mass	43.8 ± 9.9	42.1 ± 9.8	0.001
Lean body mass	46.9 ± 10.5	45.1 ± 10.5	0.001
Percent body fat	27.6 ± 9.9	28.9 ± 10.0	0.04
Fat distribution	0.86 ± 0.09	0.86 ± 0.09	0.95

Note: Data presented as mean ± standard deviation; p-value significant at p < 0.05.

To determine whether the differences elicited were influenced by participants’ sex, gender stratification was done in both groups (Table 3). No significant differences were elicited in men. In women, however, the perceived stress group had a significantly higher total cholesterol than the controls (*p* = 0.01). The rest of the variables were not significantly different from one another.

**Table 3:** Characteristics of men and women students according to the presence of perceived stress

	**Men**			**Women**		
	**Control**	**Stressed**	**P value**	**Control**	**Stressed**	**P value**
N	659	453		385	381	
Age (years)	18.4 ± 2.7	18.4 ± 2.3	0.65	17.8 ± 2.9	18.1 ± 2.1	0.18
BMI (kg/m^2^)	24.2 ± 5.9	24.6 ± 6.7	0.39	23.3 ± 4.5	23.1 ± 5.2	0.69
Waist circumference (cm)	84.7 ± 16.1	84.1 ± 19.1	0.62	73.1 ± 11.6	73.5 ± 12.4	0.66
Hip circumference (cm)	101.3 ± 15.9	101.4 ± 15.4	0.87	98.3 ± 11.4	97.9 ± 12.5	0.65
SAD (cm)	20.0 ± 6.5	20.3 ± 8.1	0.47	13.3 ± 5.2	13.4 ± 4.2	0.82
Total cholesterol (mmol/l)	4.2 ± 0.83	4.2 ± 0.86	0.77	4.4 ± 1.0	4.6 ± 1.0	0.01
Glucose (mmol/l)	5.1 ± 0.61	5.2 ± 0.60	0.85	5.0 ± 0.49	5.1 ± 0.56	0.26
HDL (mmol/l)	1.1 ± 0.25	1.1 ± 0.25	0.55	1.3 ± 0.36	1.4 ± 0.32	0.82
Triglycerides (mmol/l)	1.0 ± 0.52	1.1 ± 0.54	0.46	0.84 ± 0.37	0.87 ± 0.43	0.36
Basal metabolic rate (BMR)	1808.5 ± 243.4	1801.5 ± 250.0	0.70	1430.2 ± 146.7	1420.7 ± 161.5	0.41
Arm muscle circumference (AMC)	23.3 ± 3.1	23.4 ± 3.3	0.77	20.2 ± 0.9	20.0 ± 2.1	0.33
Body cell mass (BCM)	37.1 ± 6.4	36.7 ± 6.6	0.51	26.7 ± 3.7	26.5 ± 4.1	0.55
Intracellular measured value	26.6 ± 4.7	26.5 ± 4.6	0.72	19.3 ± 3.8	18.9 ± 2.9	0.24
Extracellular measured value	12.4 ± 2.2	12.4 ± 2.3	0.80	9.3 ± 1.3	9.2 ± 1.4	0.60
Protein mass measured value	10.7 ± 1.8	10.6 ± 1.8	0.65	7.6 ± 1.1	7.5 ± 1.1	0.48
Mineral mass measured value	3.4 ± 0.49	3.3 ± 0.49	0.46	2.6 ± 0.28	2.6 ± 0.31	0.43
Fat mass measured value	19.0 ± 13.3	19.2 ± 13.6	0.87	19.7 ± 8.7	19.9 ± 9.9	0.75
Body water	39.1 ± 6.7	39.1 ± 7.4	0.97	28.4 ± 3.9	28.2 ± 4.3	0.51
Soft lean mass	49.8 ± 8.5	49.5 ± 8.7	0.63	36.1 ± 5.1	35.9 ± 5.5	0.55
Lean body mass	53.2 ± 9.2	52.9 ± 9.5	0.70	38.7 ± 5.2	38.5 ± 5.8	0.67
Percent body fat	24.0 ± 9.9	24.1 ± 10.0	0.96	32.3 ± 7.6	32.4 ± 8.3	0.78
Fat distribution	0.87 ± 0.11	0.87 ± 0.11	0.96	0.85 ± 0.07	0.85 ± 0.07	0.57

Note: Data presented as mean ± standard deviation; p-value significant at p < 0.05.

Table 4 shows the associations of the different variables measured using the perceived stress scores as a dependent variable. Significant inverse associations, though small, were observed between perceived stress and intracellular (*p* = 0.001) and extracellular (*p* = 0.001) water measured value, as well as soft lean mass (*p* = 0.008). Percent body fat had a small but significant positive association to perceived stress (*p*-value 0.01). These associations persisted in the stress group while no associations were observed in the non-stressed group (see Table 4).

**Table 4:** Associations of different metabolic variables using perceived stress score as a dependent variable

	**Over all**		**Non-stressed**		**Stressed**	
	***r***	***p***	***r***	***p***	***r***	***p***
Age (years)			−0.006	0.86	0.001	0.98
BMI (kg/m^2^)	0.01	0.60	0.02	0.45	0.02	0.50
Waist circumference (cm)	−0.04	0.08	−0.01	0.68	−0.03	0.37
Hip circumference (cm)	−0.02	0.38	0.02	0.46	0.008	0.82
SAD (cm)			−0.008	0.80	−0.07	0.05
Total cholesterol (mmol/l)	0.06	0.01	0.01	0.65	0.04	0.25
Glucose (mmol/l)	0.01	0.61	−0.01	0.75	−0.01	0.63
HDL-cholesterol (mmol/l)	0.05	0.03	−0.01	0.65	0.03	0.48
Triglycerides (mmol/l)	0.003	0.88	−0.04	0.23	0.007	0.84
Basal metabolic rate (BMR)	−0.04	0.07	0.01	0.66	−0.03	0.38
Arm muscle circumference (AMC)	−0.02	0.41	0.01	0.56	0.03	0.40
(Body Cell Mass) BCM	−0.04	0.08	0.01	0.63	−0.04	0.25
Intracellular measured value	−0.09	0.001	0.01	0.68	−0.16	<0.001
Extracellular measured value	−0.09	0.001	0.009	0.79	−0.15	<0.001
Protein mass measured value	−0.04	0.15	0.001	0.96	−0.03	0.39
Mineral mass measured value	0.001	0.98	0.04	0.24	−0.08	0.02
Fat mass measured value	0.01	0.51	0.03	0.43	−0.03	0.32
Body water	−0.06	0.01	0.01	0.67	−0.17	<0.001
Soft lean mass	−0.07	0.008	0.001	0.97	−0.11	0.005
Lean body mass	−0.01	−0.61	0.02	0.61	−0.05	0.23
Percent body fat	0.06	0.01	0.03	0.41	0.05	0.17
Fat distribution	0.02	0.37	0.02	0.51	0.02	0.63

Note: Data presented as coefficient (R); p-value significant at p < 0.05.

## Discussion

This study highlights the high level of perceived stress among Saudi students entering pre-college education. The prevalence of high perceived stress, which was noted among 44% of students, is similar to the ones examined in Egypt and Malaysia [15,16], although these studies utilized medical students as subjects as opposed to the pre-college students in the present study. Comparatively, only total cholesterol was significantly elevated among female students with perceived stress than those without, with no apparent differences observed in male students.

The presence of perceived stress was significantly and inversely associated with several anthropometric parameters in the present study, including fat, mineral and protein values, suggesting modest metabolic alterations at this stage of life. These significant associations have clinical implications, despite the fact that the tool used to assess stress is highly subjective and dependent on the individual’s personal judgment of how stressed he or she is. It has been well established that stress, both acute and chronic induces a powerful cascade of metabolic and inflammatory processes [17]. How the students in question respond to stress may affect their development and largely predispose to various endocrine, metabolic, autoimmune and psychiatric disorders [18].

The start of college life coincides with emerging adulthood, when young people begin to take control of their own eating and exercise behaviors [19,20]. Both the level and duration of stress encountered during this vulnerable period may, thus, further contribute to dysregulation of the stress system, subjecting adolescents to a vicious cycle between distress and distorted self-image, maintaining and worsening distress and associated physiologic and somatic changes [21-23].

We have previously observed that abnormal metabolic patterns among Arabs are highly heritable and can manifest as early as preadolescence [24]. Furthermore, we demonstrated that abnormal sleeping patterns and micronutrient deficiencies such as calcium and vitamin D can increase the likelihood of weight gain in Arab adolescents, a finding also observed in the present study [25,26]. These past observations, and the findings of the present study, highlight the importance of chronic disease prevention by targeting the younger population. It is worthy to note that while the Preparatory Year of King Saud University is replete with several physical education classes, its food court is not as health-conscious as it should be. Implementing school-based health promotion that has been proven efficacious in the prevention of childhood obesity [27], therefore, should be taken into consideration.

The strengths of this observational study are that it contributes to the literature in significant ways. First, it takes into account findings from an understudied population from an equally understudied ethnic group. Second, the gender difference elicited in terms of cardiometabolic risk factor prevalence showcase the need for a tailored approach in the prevention of cardiometabolic manifestations in this vulnerable age group. Nevertheless the authors also acknowledge several limitations. The cross-sectional and single center approach despite the big sample size limits the causality and generalizability of the study findings.

## Conclusion

In summary, young Arab adults entering college, who perceive themselves as stressed, are already at increased cardiometabolic risk and this vulnerability appears more prominent in females than males. A follow-up interventional study is needed to validate the present findings and to determine whether stress management and increased coping will translate to an improved cardiometabolic profile among young Arab adults.

## Competing interest

The authors declare that they have no competing interests.

## Authors’ contributions

NMA, AA and AMA conceptualized the study. OSA, KMA and MSA participated in interpreting data and in drafting and revising the final manuscript. SS and GPC interpreted data and drafted the final manuscript. All authors read and approved the final manuscript.

## Pre-publication history

The pre-publication history for this paper can be accessed here:

http://www.biomedcentral.com/1471-2458/14/391/prepub
